# Organic Contaminants and Interactions with Micro- and Nano-Plastics in the Aqueous Environment: Review of Analytical Methods

**DOI:** 10.3390/molecules26041164

**Published:** 2021-02-22

**Authors:** Julia Reichel, Johanna Graßmann, Oliver Knoop, Jörg E. Drewes, Thomas Letzel

**Affiliations:** 1 Urban Water Systems Engineering, Technical University of Munich, Am Coulombwall 3, 85748 Garching, Germany; julia.reichel@tum.de (J.R.); j.grassmann@tum.de (J.G.); oliver.knoop@tum.de (O.K.); jdrewes@tum.de (J.E.D.); 2 Analytisches Forschungsinstitut für Non-Target Screening GmbH (AFIN-TS GmbH), Am Mittleren Moos 48, 86167 Augsburg, Germany

**Keywords:** sorption, microplastic, nanoplastic, analytic methods, GC, HPLC

## Abstract

Micro- and nanoplastic particles are increasingly seen not only as contaminants themselves, but also as potential vectors for trace organic chemicals (TOrCs) that might sorb onto these particles. An analysis of the sorbed TOrCs can either be performed directly from the particle or TOrCs can be extracted from the particle with a solvent. Another possibility is to analyze the remaining concentration in the aqueous phase by a differential approach. In this review, the focus is on analytical methods that are suitable for identifying and quantifying sorbed TOrCs on micro- and nano-plastics. Specific gas chromatography (GC), liquid chromatography (LC) and ultraviolet-visible spectroscopy (UV-VIS) methods are considered. The respective advantages of each method are explained in detail. In addition, influencing factors for sorption in the first place are being discussed including particle size and shape (especially micro and nanoparticles) and the type of polymer, as well as methods for determining sorption kinetics. Since the particles are not present in the environment in a virgin state, the influence of aging on sorption is also considered.

## 1. Introduction

Micro- and nanoplastic particles can serve both as sources and sinks for pollutants in the environment. Therefore, on the one hand, sorption of pollutants on microplastics might pose a problem; on the other hand, microplastic itself must be considered as a contaminant. Monomers, additives, plasticizers, and others can desorb and may cause additional potential risks [1,2,3,4,5,6,7].

In recently published reviews, sorption of trace organic chemicals (TOrCs) on micro- and nanoplastics has already been examined in detail [8,9,10,11]. In these studies, the focus is on the influence of the physical properties of particles (such as size, surface, crystallinity) and the resulting interaction properties of TOrCs and polymers. The sorption mechanisms and ecotoxicological factors are also considered. In contrast to the previously published articles, the purpose of this article is to provide an overview of suitable analytical approaches for determining the sorption of TOrCs on particles. The focus is on GC, HPLC, and UV-VIS methods. Furthermore, typical sorption strategies will be discussed. 

Concerns about the possible harmful effects of microplastics relates not only to the particles themselves but also to their ability to transport pollutants. Those pollutants can be divided into two groups: (i) hydrophobic chemicals adsorbed from the aquatic environment due to their affinity for the hydrophobic surface of plastics and (ii) additives, monomers, and oligomers present as constituents of polymers [12]. Adsorbed hydrophobic pollutants with low water solubility become more mobile indirectly by binding to plastic particles. Thus, their transport on particles can increase their distribution in environmental matrices and organisms as well as their bioavailability.

The sorption processes depend mainly on the nature of the polymer and can be divided into adsorption to the surface or absorption into the polymer [5,13,14]. The principle of both sorption types is to achieve an equilibrium of TOrCs concentrations between the solid and liquid phases. The sorption equilibrium can be reached either quickly by adsorption (onto particles) or slower by absorption (into the particle structure) [15]. In recent years, the analysis of pure micro- and nanoplastics has gained great interest but also the analysis of sorbed TOrCs on these particles [8,16]. With optical analysis methods like Raman spectroscopy or Fourier-transform infrared spectroscopy (FTIR) analysis, quantitative analyses of particles and analyses of particle size as well as shape can be conducted [17,18]. Thermal analysis methods such as pyrolysis gas chromatography-mass spectrometry (Pyr-GC/MS) can be used to analyze TOrCs or the additives in the polymers [19,20]. This review summarizes the current state-of-the-art in analyzing the degree of TOrCs sorption on micro- and nanoparticles.

## 2. Analysis of TOrCs on Micro- and Nanoplastic Particles: Typical Methods and Techniques

Analysis of sorbed TOrCs on particles in a liquid phase can be performed in either the aqueous or gas phase, or on the particles, respectively [5,13,20,21]. Different techniques like GC/MS, HPLC-DAD, UHPLC-MS/MS, UV spectrometer, or liquid scintillation counting are commonly used for this purpose [21,22,23,24]. 

### 2.1. General Experimental Design of Sorption Experiments

**Figure 1:**
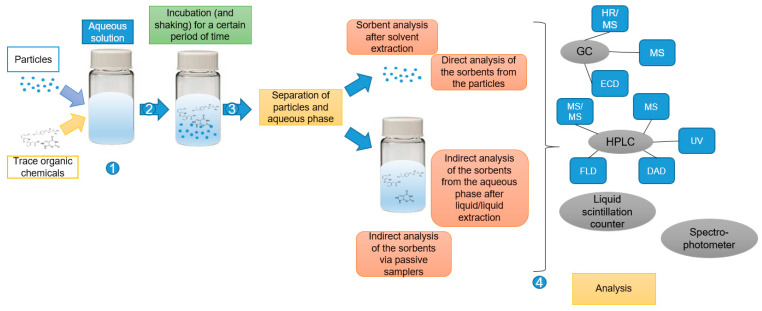
Workflow from a sample preparation of sorption experiments for analytical detection techniques. First, the particles and the TOrCs are incubated in aqueous solution for a certain period of time. This is followed by the separation of the particles and the aqueous phase. Depending on the choice of analytical technique, either the aqueous or the particle phase is processed. The analysis is either based on a gas chromatography method followed by high resolution/mass spectrometry (HR/MS), mass spectrometry (MS) or electron capture detection (ECD) analysis or based on a high performance liquid chromatography (HPLC) method followed by MS/MS, MS, ultraviolet (UV), diode-array detector (DAD) or fluorescence detector (FLD). Further analytical methods are liquid scintillation counter and spectrophotometer.

Principally, the experimental design studying sorption kinetics or processes in aqueous suspensions is identical in all experimental approaches. Selected particles and TOrCs are added to a liquid phase such as ultrapure water, freshwater, sea water, or synthetic water containing humic acids to mimic natural organic matter [13,20,25]. Variations in pH, salinity, or humic substances are made to simulate different environmental conditions [26,27,28]. Some studies use microparticles, additive-free particles, or extracted particles from cosmetics covering a certain size range [29,30]. Commonly targeted TOrCs used as sorbates in these investigations represent antibiotics, additives, pesticides, biocides, endocrine disrupting chemicals, hormones, or disinfection byproducts [24,31,32,33,34,35,36]. A schematic of the sample preparation is shown in Figure 1. In the aqueous solution, particles and TOrCs are incubated for a certain period of time and the suspension is subsequently shaken for various time periods (Table 1 and Table 2). Subsequently, the particles must be separated from the aqueous phase for analysis. This is achieved for instance by filtration or centrifugation [20,37,38]. The analysis of the sorbed substances happens either directly on the particles or indirectly by solvent extraction (e.g., n-hexane, dichloromethane) decoupled from the particles [13,20,39,40]. An indirect analysis of the aqueous phase via liquid/liquid extraction or by a passive sampler is also possible [4,21,41,42]. Due to the remaining concentration in the aqueous phase, an assessment can be made on the amount of sorbed substances on the particles [43]. Furthermore, the gas phase can also be investigated [5,44].

The final analysis can be performed by various analytical techniques, such as GC/MS, GC/ECD, HPLC/MS, HPLC/UV, liquid scintillation counter, or spectrophotometer [4,6,24,30,45,46,47,48,49]. An overview of the workflow from a sample preparation for the analytical technique is illustrated in Figure 1. A detailed consideration of the different analytical techniques is presented in the following sections.

### 2.2. GC/MS

With GC/MS, volatile substances can be determined. This method is often used for the investigation of sorbed substances on polymer particles [13,26,45,46]. In a GC/MS analysis, either the aqueous phase, the particles, or the gas phase can be examined for TOrCs [5,13,39]. An overview of different sorption studies of microplastic particles performed with GC/MS is shown in Table 1. The advantages of GC analysis are that it allows direct analysis of TOrCs from the particle, requires little sample preparation, and is, thus, quick to perform (2–3 h per sample) [20,50,51]. By coupling with, e.g., a pyrolysis unit, polymers can be identified in addition to sorbed TOrCs [50]. In combination with an MS, detailed results for target and non-target analysis can be provided.

#### 2.2.1. Direct Analysis by Pyrolysis and TED-GC/MS

Currently, GC/MS analysis is mainly used for microplastic analysis and polymer identification. For this purpose, the GC/MS is coupled with a pyrolysis unit (Pyr) or a thermal-extraction-desorption (TED)/thermogravimetric (TGA) unit [19,50,51,52,53,54]. A recently published review provides a good summary of these methods [55]. In general, only the polymer can be identified and quantified with the thermoanalytical systems. A determination of size is not possible. An established method for the identification of plastics in various environmental matrices is Pyrolysis-GC/MS. [52,56,57,58,59,60]. One advantage of this method is that both micro- and nanoplastics can be analyzed [61]. Recently, pyrolysis methods have been developed further by coupling with for instance a sequential pyrolysis, a double shot pyrolysis, or a thermal desorption (TD) unit with pyrolysis [19,20,50,53]. The aim of these methods is to identify not only the polymer itself but also additives or sorbed substances. 

Double shot pyrolysis can be run in two different modes [53]: (1) in desorption mode, the volatile substances such as additives are desorbed; (2) in pyrolysis mode, the polymers are degraded. In sequential pyrolysis, several runs with different temperature maxima are performed in series [50].

TD-Pyr-GC/MS (thermodesorption-pyrolysis-GC/MS) combines these two systems into one analytical setup to investigate sorbed TOrCs before analyzing the polymer [20]. After method development, the TOrCs can be identified in the first step and the polymer type in the second step. 

Another method for the determination of polymers and additives is via Thermal-Extraction-Desorption-GC/MS (TED-GC/MS) coupled with thermogravimetric analysis (TGA) [54]. In TGA, the polymers are heated and their volatile decomposition products are trapped on a solid-phase adsorber such as a Gerstel Twister or a Sorb-Star^®^ [62,63]. The trapped substances are heated and then analyzed by GC/MS. An advantage of this method is that the number of particles can also be quantified. 

The benefit of these thermoanalytical systems is that the samples can be analyzed in a short amount of time (2–3 h) [20]. There is no need for complex extraction procedures, but the samples can usually be analyzed directly. Often, however, there is a carry-over of the samples during the Pyr-GC/MS [64]. Therefore, it is recommended to run many blanks to identify and eliminate them. Analytical reproducibility can also be problematic [64].

#### 2.2.2. GC/MS Analysis after Extraction of Sorbents

For indirect analysis of sorbed substances or additives, these can be extracted from the microplastic particles with solvents, see Table 1. Table 1 shows the phase that is analyzed to obtain the concentrations of TOrCs. For this purpose, the trace organic chemical can be extracted from the particle, for instance by means of soxhlet extraction [65]. The TOrCs are washed off the particle with a solvent (e.g., dichloromethane), concentrated, purified, and finally analyzed. Another possibility is the indirect determination of the concentration via the aqueous phase [21,41]. Therefore, a liquid/liquid extraction is performed. A portion of the aqueous solution is mixed with a solvent (e.g., hexane), placed in an ultrasonic bath and then stirred. The upper solution layer is then used for the GC/MS analysis [41]. 

### 2.3. HPLC

In addition to GC, high performance liquid chromatography (HPLC) is also an established chromatographic method for determining the sorption of TOrCs on micro- and nanoparticles [67,68,69]. Samples are usually filtered prior to analysis and the concentration on the particles is then determined indirectly via the supernatant. 

Another method is solvent extraction of the particles or solid phase extraction. The HPLC is typically coupled to a UV-detector (UV), a diode array detector (DAD), a fluorescence detector (FD), or a mass spectrometer [38,47,67,70]. A summary of selected studies performed by HPLC is shown in Table 2. In most studies, chromatography was performed with a C-18 column, which captures nonpolar to intermediate polar TOrCs [22,28,34,38,48,67,70,71,72,73,74,75,76]. Advantages of an HPLC analysis are that an individual separation of the individual molecules takes place and detailed results can be (re)produced [47,70,71].

### 2.4. Further Analytical Techniques for the Determination of Sorbed TOrCs

Besides the GC and LC methods for the identification of sorbed substances, other methods such as ultraviolet/visible spectroscopy (UV/VIS) or spectrophotometers can also be used [30,80,81], see Table 3. Here, a direct analysis of the sorbed substances is not possible; therefore, the supernatant is analyzed. During UV/VIS analysis, the concentrations of the pollutants were calculated from their absorbance. The advantages of UV-VIS analysis are the robustness of the system, the easy handling, the short measuring times, and that it is available in most laboratories. 

Measurement by liquid scintillation counting is another method for analyzing sorbed TOrCs in laboratory experiments [6,24,25,26,27,28,29,30,31,32,33,34,35,36,37,38,39,40,41,42,43,44,45,46,47,48,49,50,51,52,53,54,55,56,57,58,59,60,61,62,63,64,65,66,67,68,69,70,71,72,73,74,75,76,77,78,79,80,81,82,83,84]. For this purpose, a ^14^C labeled standard of the trace substance is used. The concentration of the trace compound is then determined by counting the decay of the ^14^C trace compound using liquid scintillation counting.

## 3. Analysis of TOrCs on Micro- and Nanoplastic Particles: Typical Sorption Strategies

In the initial microplastic studies, the focus was mainly on detection in environmental systems to get an overview of the distribution of the plastic [84,85]. A detailed review by Li et al. (2019) summarizes the occurrence of microplastics in freshwater systems in terms of microplastic sources, distribution, sampling, and processing methods, as well as polymer characterization [86]. The difficulties of qualitative and quantitative analyses are also addressed. In order to investigate the distribution of micro- and nanoplastics in general, numerous studies have been conducted in fresh water and salt water [87,88,89,90,91]. In these studies, the analysis of the particles was performed using Raman and µFTIR.

Recently, many reviews have been published dealing with interactions between plastic particles (micro and nano) and TOrCs [8,9,11,92,93,94,95]. Firstly, the plastic itself is examined more closely, including polymer type, the specific surface of the particles with their functional groups and physicochemical properties, crystallinity, polarity, and additives [8,16,55,92,96]. The factors of the surrounding matrix, such as salinity, pH, dissolved organic matter (DOM), coexisting organic contaminants, and ionic strength [9,93] are also considered. The main retention mechanisms from TOrCs to micro- and nanoplastics are pore filling, hydrophobic hydrogen bonding, π-π-, electrostatic interactions, and van der Waals forces [9]. A further important issue is the ageing of polymers and the resulting influence on the sorption of TOrCs [97]. Finally, the influences of the plastic and TOrCs are examined regarding their toxicological relevance for the aquatic environment and the possible impact on human health [11,94,95]. 

### 3.1. Strategies Characterizing the Polymer Type

Several methods are available to identify the polymer type of the plastic particle, such as physical characterization (e.g., microscopy) and chemical characterization (e.g., Fourier transform infrared (FTIR) and Raman spectroscopy) and were summarized in a recent review by Shim et al., 2017 [16]. Spectroscopic analysis of polymers requires purification and isolation of environmental samples [97]. FTIR and Raman spectroscopy are non-destructive and the polymer type can be determined with the help of a database [16]. Thermoanalytical methods such as Pyr-GC/MS, TED-GC/MS can be used to identify and quantify polymers by their characteristic products, presented by La Nasa et al. (2020) and Yakovenko et al. (2020) [55,96]. Thermoanalytical methods have no size limitations and several polymer types can be identified in parallel. However, a minimum amount of polymer is required. For example, a minimum of 10 mg is required for a TED-GC/MS analysis or 60 µg for a Pyr-GC/MS analysis [52,54]. 

Main characteristics affecting sorption on a plastic particle are crystallinity, density, structure, hydrophobicity, and the glass transition temperature T_G_. These factors were discussed comprehensively in detail in a recently published short review [8]. In a study comparing the sorption of different polycyclic aromatic hydrocarbons (PAHs) with low-density polyethylene (LDPE) and high-density polyethylene (HDPE), the density of the polymers was observed to have a negative effect on the sorption rate. The sorption capacity decreased with increasing density of the polymers used [14]. However, the density of polymers with crystalline and amorphous components, such as HDPE, was determined by the ratio of crystallinity. In amorphous materials, the hydrophobic bonds are less stable than in crystalline materials [98]. Since only the amorphous fraction can dissolve substances, polymers with a high crystallinity content should have a limited absorption capacity [99,100]. The amorphous region within polymers can be classified as either glassy or rubbery, which is also an indication of sorption capability [12]. The surface appearance is also important for sorption processes. Napper et al. (2015) showed that rough polyethylene (PE) microplastic particles adsorbed more DDT and phenanthrene than smooth ones [29]. The crystallinity of polymers can be measured by X-ray diffraction [101].

Notably, desorption hysteresis was only observed for nonpolar/weakly polar contaminants, likely because nonpolar compounds tended to adsorb in the inner matrices of glassy polymeric structure of polystyrene (resulting in physical entrapment of adsorbates), whereas polar compounds favored surface adsorption [32]. The glass transition temperature (T_g_) defines whether a polymeric rubber-like or glass-like material is present. The T_g_ can be determined by using a thermogravimetric differential scanning calorimetry analyzer (TG-DSC) [73]. Rubber-like polymers are normally above their T_g_ values if they are not plasticized. At room temperature, this results in greater flexibility, which facilitates sorption of impurities. Glassy polymers are usually below their T_g_ and are also referred to as condensed (glasslike) [2]. In general, rubbery polymers (such as HDPE, LDPE, or PP) are expected to allow greater diffusion of impurities into the polymer than glassy polymers (such as polyethylene terephthalate (PET) or polyvinyl chloride (PVC)) [2,3]. However, there are exceptions, such as polystyrene (PS). The average sorption capacity is higher than the T_g_ predicts [1,2,4,5]. A possible explanation for this is the presence of benzene. The phenyl group increases the distance between the polymer chains and can facilitate adhesion and integration of impurities into the polymer [2,5]. However, when comparing polyethylene (PE) and PS in the adsorption and desorption of triclosan, a higher sorption rate was found on PE particles. Triclosan also desorbed faster from the PE particles [34].

A summary of studies in which the sorption capacity of TOrCs and their mechanisms were tested on different particle types is shown in Table 4. Here, reference particles were used in all experiments and were, therefore, not further analyzed in any of the studies.

### 3.2. Strategies Characterizing Particle Size and Shape (Micro vs. Nano)

Micro- and nanoplastic particles may be derived from fragmentation of larger plastic items by means of photolytic, mechanical, and biological degradation without significant chemical degradation [85,102,103]. Thereby, microplastic particles can further disintegrate into nanoplastics [104,105,106]. As the surface area increases with decreasing particle size, it is assumed that smaller particles are of greater ecotoxicological relevance since the capacity for adsorption of TOrCs increases. 

Typical methods for particle sizing can be performed by microscope, FTIR, and Raman spectroscopy [16]. However, there are also limitations. A determination with an optical microscope is often only possible up to 100 µm because smaller particles can also consist of sediment particles [107]. No distinction is then possible using an optical microscope. A FTIR analysis is possible up to 10 µm, a Raman analysis is limited to 100 nm [108,109]. The relationship between particle, surface size, and sorption capacity is considered in more detail in the recently published review by Wang et al., 2020 [9].

In a study by Li et al. using different PS microparticles, it was shown that the sorption capacity of triclosan increases with decreasing particle size of PS [72]. During sorption experiments with micro- and nanoplastic particles, aggregation must also be taken into account. This can happen between two similar (homoaggregation) or two different (heteroaggregation) particles [1]. Aggregation is generally controlled by the ionic strength and valence of the electrolytes in the surrounding media; however, the polymer coating of the particles may also play a role [1,110]. Wang et al. (2019) also showed that the sorption of phenanthrene on the particles was reduced by aggregation of the particles [37]. It has also been shown that nanoparticles agglomerate more and, thus, the specific surface area is reduced again, which can lead to low sorption [37]. A new study by Sun et al. (2020) shows that the agglomeration is strongly dependent on the surrounding matrix [111]. Nanoplastic particles are stable in fresh water due to the Brownian motion and structural layer force, but aggregate in brackish or seawater. In a study with nano-PS, however, it was also shown that the aggregation of the nanoparticles does not change the sorption capacity [70]. The sorption isotherms were the same for aggregated and non-aggregated particles. This indicates that the TOrCs were reaching the sorption sites on the original nanoparticles regardless of the aggregation state. In order to enable comparison of data, the methods for detection, analysis and toxicological assessment of nanoplastics, which are currently still in their initial stages, must first be improved [112]. 

### 3.3. Strategies Characterizing Weathered/Aged Particles

Factors that can influence the aging of plastics are, e.g., UV-radiation, temperature, salt content in the environment, and biofilm formation [113]. These causes the plastic to break into smaller and smaller pieces and additives can be released. Induced aging of particles can be carried out for instance by Photo-Fenton oxidation, UV-irradiation, or microbial degradation [79,97].

Investigating aged and unaged microplastic particles, scanning electron microscopy (SEM), transmission electron microscopy (TEM), and FTIR can be used to determine the physical dimensions, morphologies, and chemical compositions [73,78,101,114]. The specific surface area and micropore volume can be studied with an accelerated surface and porosimetry system (ASAP) [83].

Differences in the sorption of aged particles compared to untreated particles are evident in the sorption mechanisms. It was shown that the adsorption of TOrCs in untreated PS particles is based on π-π interaction, whereas in aged PS particles, electrostatic interaction and hydrogen bonding prevail [78]. The results of this study indicated that aging of PS significantly changed the adsorption behavior via the changes of oxygen-containing functional groups and specific surface area. Considering aged and non-aged PP particles in combination with the trace substance triclosan, the aged ones have a higher adsorption capacity than pure microplastics [73]. The sorption affinity was increased with the increase of ionic strength. Study results suggest that particles exposed to weathering processes and the simultaneous presence of several organic trace compounds may affect the biological ecosystem in the natural environment [79]. This is in contrast to a study by Koelmans et al. (2016) on microplastics and hydrophobic organic chemicals (HOCs). The authors conclude that more HOCs accumulate in natural prey and, thus, the risks from microplastics are not increased [10]. Aged PS particles are shown to generally exhibit higher levels of oxygenated functionality with lower surface hydrophobicity than unaged particles, which also influences the sorption capacity [79]. Due to the UV aging of the particles, the surface becomes rougher. In a study by Fan et al. (2021), it was shown that the surface area of PVC particles increased by 1.85 times and that of PLA by 2.66 times [31]. At the same time, the zeta potential decreased and the adsorption capacity of the particles increased due to the aging process. Charge neutralization is one of the important mechanisms of adsorption. Studies show that the surface charge of the adsorbent is closely related to its ability to absorb pollutants [31,115,116].

### 3.4. TOrC–Microplastics Sorption and Desorption Kinetics

For the determination of the ad- and absorption kinetics, the above-mentioned analytical approaches can be applied in general [33,67,77]. However, there can be some limitations for smaller particles concerning the sampling frequency, since filtration for the separation of particles from the liquid phase requires longer periods of time with decreasing size of the investigated particles. Hence, if the analytical method requires a separation by filtration, such a limitation needs to be considered in the experimental design, especially for particles in the sub-micrometer range. Studies either did not report such limitations since the particles were either too big (>1 µm) to encounter the problem [9,30,39,48,76,77], or a filtration step was avoided, i.e., by negligible depletion solid phase extraction, the aqueous boundary layer permeation method, head space extraction techniques, or else [4,5,32,82]. 

For the investigation of the desorption kinetics, another issue needs to be overcome. Since the equilibrium of the sorption mechanism is mostly on the side for the polymer phase, especially for hydrophobic compounds, low aqueous concentration TOrCs must be expected and slow desorption kinetics must also be assumed [117,118]. For hydrophobic compounds, a third phase can be included since TOrCs-sink within the experimental design, such as either virgin polymer particles or another sorptive phase, such as solid-phase microextraction. Here, the sorbent acting as sink should be available in excess to ensure the desorption from the loaded polymer particles as the limiting step [32,117,119].

A desorption hysteresis is reported to be higher for hydrophobic compounds than for polar compounds, but also depends strongly on the polymer. For PE [118], and glassy polymeric domains of PS [32], a significant hysteresis for hydrophobic compounds has been reported. Therefore, desorption of hydrophobic compounds even within more complex matrices such as gut fluids is more unlikely than for polar compounds [120]. The desorption hysteresis is also a critical parameter of TOrC–polymer interaction concerning bioaccessibility and, therefore, the environmental impact [10]. 

## 4. Conclusions and Outlook

Established analytical methods for the determination of micro- and nanoplastic particles are FTIR, Raman spectroscopy, and thermal methods such as TED-GC/MS and Pyr-GC/MS. FTIR and Raman spectroscopy can be used to identify polymers, but these methods are limited in size [52,54,108,109]. 

Considering sample preparations for the generation of sorption kinetics, properties, or processes, the experimental set-up is in most cases the same: the selected particles are incubated with the defined TOrCs over a defined period of time. Afterwards, the aqueous phase is separated from the particle phase. The following analysis of the sorbed TOrCs can be performed either via the filtrate or the particles.

For a simple and fast target analysis of TOrCs in the supernatant, a UV-VIS method is recommended, since this is easy to use and is available in most laboratories. Specific absorbance of individual trace compounds can be determined. However, this method is not as sensitive as separation coupled detections, such as with HPLC and GC. Using HPLC coupled with a UV, MS, FD, or DAD, this technique provides more detailed results because the molecules can be separated individually. Direct trace analysis of the particle is not possible with either UV-VIS or HPLC methods. The most established method for polymer analysis is a GC based one. By coupling specific systems such as TED-GC/MS, TD-Pyr-GC/MS, double shot pyrolysis, or sequential pyrolysis the possibility is even offered to perform a direct TOrCs analysis of the particles followed by polymer analysis. 

Considering future research, the focus should be mainly on the following points:

(1) Up to now, either the sorbed TOrCs on the particles or the supernatant have only been analyzed. For the preparation of a mass balance, a complete analysis of particles and aqueous phase would be interesting.
(2) In most conducted studies, the TOrCs are individually adsorbed onto the polymer. However, it is not to be expected that TOrCs will occur individually in the environment, but are present in mixtures. Napper et al. (2015) and Velzeboer et al. (2014) investigated the competitive sorption of phenanthrene and DDT on PE and PVC, respectively, and both found that DDT sorbed slightly more than phenanthrene [29,45]. Future studies should focus more on how TOrCs affect each other regarding sorption strength and capacity.
(3) The largest challenge in the analysis of TOrCs on micro- and nanoplastic particles will certainly be the removal of inorganics and larger organics such as biofilms without adversely affecting the sorbed TOrCs.

## Figures and Tables

**Table 1:** Summary of GC/MS methods to analyze sorbed substances on various microplastic particles like polyethylene (PE), polystyrene (PS), polyamide (PA), polyvinyl chloride (PVC), polypropylene (PP), polyethylene terephthalate (PET) and polymethyl methacrylate (PMMA).

Particle Type	Particle Size (µm)	Sorbate	Analytical Method	Analyzed Phase	Reference
PE	260	Phenanthrene, Tonalide, Benzophenone	GC/MS after extraction with cyclohexane	Particle (Extraction)	[13]
PE, PS	PE: 260, PS: 250	Atrazine, Benzotriazole, Caffeine, Carbamazepine, Carbendazim, DEET, Diazinon, Diclofenac, Ibuprofen, MCPA, Mecoprop, 4-Nonylphenol, Phenanthrene, Propiconazole, Tris(2-chloroisopropyl)-phosphate (TCPP), Tebuconazole, Terbutryn, Torasemide, Triclosan	GC/MS, LC-MS/MS after extraction with cyclohexane	Particle (Extraction)	[26]
PA, PE, PVC, PS	<250	n-Hexane, Cyclohexane, Benzene, Toluene, Chlorobenzene, Ethylbenzoate, Naphtalene	Headspace GC/MS or in-tube-microextraction	Gaseous phase	[5]
PS (aged)	125–250	Various aliphatics and aromatics	GC/MS headspace from three-phase system	Gaseous phase	[44]
PE, PS, Fullerene, Sediment	PE: 10–180 PS: 0.07	17 Polychlorinated biphenyls (PCBs)	GC/MS after extraction with pentane-dichloromethane	Aqueous phase via passive sampler	[45]
PE, PP, PS	320–440	8 Polycyclic aromatic hydrocarbons (PAHs), 4 Hexachlorocyclohexanes (HCHs), 2 Chlorinated benzenes (CBs)	GC-ECD after extraction with n-hexane	Aqueous phase and PDMS phase	[4]
PP	450–850	Tonalide, Musk xylene, Musk ketone	GC/MS after extraction with n-hexane and dichloromethane	Particle (extraction)	[46]
PS, PE, PET	PE: 3–16 PS:10 PET: <300	38 PCB congeners	GC-HRMS after soxhlet extraction with dichloromethane	Particle (extraction)	[65]
PE, PP (environmental samples)	<500	PCBs (IUPAC nos. 28, 52, 101, 118, 138, 153, 180)	GC-ECD after soxhlet extraction with dichloromethane	Particle (extraction)	[66]
PS	2; 1; 0.1	Eighteen unsubstituted hydrophobic organic chemicals (HOCs)	GC/MS after liquid / liquid extraction	Aqueous phase via passive sampler	[41]
PE, PS, PVC	<150	Five polyhalogenated carbazoles (PHCs)	GC/MS after washing with n-hexane and dichloromethane	Particle (extraction)	[39]
PE, PP, PS	100–150	9-Nitroanthracene	GC/MS after liquid/liquid extraction	Aqueous phase	[21]
PP	450–850	3,6-Dibromocarbazole and 1,3,6,8- Tetrabromocarbazole	GC/MS after extraction with n-hexane and dichloromethane	Particle (extraction)	[40]
PS, PE, PMMA	PS: 40, 41, 0.078 PMMA: 48 PE: 48	Phenanthrene, Triclosan, α-Cypermethrin	TD-Pyr-GC/MS	Particle (directly)	[20]

**Table 2:** Summary of HPLC methods to analyze sorbed substances on micro- and nanoplastic particles like polybutylenadipat-terephthalat (PBAT), low-density polyethylene (LD-PE) or high-density polyethylene (HD-PE).

Particle Type	Particle Size (µm)	Sorbate	Analytical Method	Analysis	Reference
PS	0.5, 0.235, 0.80, 30, 50, 102, 170	Phenanthrene, Nitrobenzene	HPLC	Supernatant	[37]
PE, PP, PS, PVC	<200	Tylosin	HPLC + DAD	Supernatant	[67]
PA, PE, PET, PS, PVC, PP	100, 150	Sulfamethoxazole	HPLC	Supernatant	[77]
PBAT, PE, PS	PBAT: 2338 ± 486, PE: 2628 ±623/Reference Particles: PE: 400PS: 250	Phenanthrene	HPLC + -UV	Supernatant	[38]
PE, PS, soil	PE: 225 ± 41 PS: 313 ± 48	Triclosan	HPLC + UV	Methanol extraction of the particles	[34]
PE, PS, PP, PA, PVC	75–180	Sulfadiazine, Amoxicillin, Tetracycline, Ciprofloxacin, Trimethoprim	HPLC + UV	Supernatant	[71]
PS	75.4, 106.9, 150.5, 214.6	Triclosan	HPLC + UV	Supernatant	[72]
PS	0.07	Phenanthrene, Anthracene, Fluoranthene, Pyrene, Benzo[a]anthracene, Chrysene, Benzo[b]fluoranthene, Benzo[k]fluoranthene, Benzo[a]pyrene, Benzo [g,h,i] perylene	HPLC + FD	Extraction via Polyoxymethylene sheets	[70]
PS (aged)	50.4 ± 11.9	Atorvastatin, Amlodipine	HPLC + UV	Supernatant	[78]
PET	<150	4-Chlorophenol, 2,4,6-Trichlorophenol, Fulvic acid	HPLC + UV	Supernatant	[48]
PP (aged)	<180	Triclosan	HPLC + UV	Supernatant	[73]
PP, LD-PE, HD-PE, PVC	63–125	Enrofloxacin, Ciprofloxacin, Norfloxacin, 5-Fluorouracil, Methotrexate, Flubendazole, Fenbendazole, Propranolol, Nadolol	HPLC + DAD	Supernatant	[74]
PS (weathered)	139–207	4-Hydroxybenzophenone, Benzophenone-1, ethylhexyl methoxycinnamate, Octocrylene	UHPLC + S/MS	Supernatant	[79]
PVC, PLA	PLA: 250–550 PVC: 75–150	Tetracycline, Ciprofloxacin	HPLC	Supernatant	[31]
nano-PS, carboxyl-functionalized polystyrene nano-PS-COOH	Nano-PS: 0.05 Nano-PS-COOH: 0.055	Norfloxacin, Levofloxacin	HPLC + FD	Supernatant	[75]
PE, PS, PP	<280	Tetracycline	HPLC + FD	Supernatant	[22]
PE	250–280	Carbamazepine, 4- methylbenzylidene camphor, Triclosan, 17α-ethinyl estradiol	HPLC + PAD (Solid phase extraction)	Supernatant	[28]
PE	150	Sulfamethoxazole	HPLC + UV	Supernatant	[76]

**Table 3:** Summary of UV/VIS spectroscopic methods to analyze sorbed substances on micro- and nanoplastic particles.

Polymer Type	Particle Size (µm)	Sorbate	Analytical Method	Analysis	Reference
PVC	<1.74	Triclosan	UV/VIS (282 nm)	Supernatant	[30]
PVC, PP, PS, PE	<1000	Co-existing surfactants	UV/VIS (665, 618, 627, 546, 224 nm)	Supernatant	[80]
PE	710–850	Imidacloprid, Buprofezin, Difenoconazole	UV Spectrophotometer	Supernatant	[81]

**Table 4:** Sorption capacity of different polymers. The following criteria were considered for study selection: particles should be approximately the same size and sorption mechanisms and capacities should be addressed.

Polymer Type	Sorbate	Sorbate Analytics	Sorption Capacity	Mechanisms	Reference
PE, PP, PS, PVC	Tylosin	HPLC + DAD	PE < PP < PS < PVC	electrostatic interactions, surface complexation and hydrophobic interactions	[67]
PE, PS, soil	Triclosan	HPLC + UV	PE > PS = soil	PS: π-π interactions, PE: liquid-film and intra-particle diffusion	[34]
PE, PS, PP, PA, PVC	Sulfadiazine, Amoxicillin, Tetracycline, Ciprofloxacin, Trimethoprim	HPLC + UV	PA > PS, PP, PVC, PE	Polar–polar interactions	[71]
PS, PP, PE	Tetracycline	HPLC-FD	PS > PP > PE	Polar interactions, π-π interactions	[22]
PE, PP, PVC	3,6-dibromocarbazole, 3,6-dichlorocarbazole, 3,6-diiodocarbazole, 2,7-dibromocarbazole, 3-bromocarbazole	GC/MS after washing with n-hexane and dichloromethane	PVC >> PP, PE	Intraparticle, film diffusion	[39]
PE, PS, soil	Triclosan	HPLC + UV	PE > PS = soil	PE: hydrophobic interactions PS: π-π interactions	[34]

## Data Availability

Data is contained within the article.
